# Two Cases of Early-Onset Asymptomatic Pulmonary Embolism Following Arthroscopic Rotator Cuff Repair

**DOI:** 10.7759/cureus.92167

**Published:** 2025-09-12

**Authors:** Shotaro Teruya, Takeshi Makihara, Kazuhiro Ikeda, Shinzo Onishi, Katsuya Aoto

**Affiliations:** 1 Department of Orthopedic Surgery, Institute of Medicine, University of Tsukuba, Tsukuba, JPN; 2 Department of Orthopaedic Surgery, Kasumigaura Medical Center, Tsuchiura, JPN

**Keywords:** arthroscopic rotator cuff repair, asymptomatic, d-dimer, deep vein thrombosis (dvt), early onset, lateral decubitus position, patient-controlled analgesia, pulmonary embolism (pe)

## Abstract

Arthroscopic rotator cuff repair is a widely used minimally invasive surgical procedure, and venous thromboembolism after this surgery is considered uncommon compared to lower extremity operations. Nevertheless, rare but serious events may occur, and asymptomatic cases can be easily overlooked. We present two elderly female patients who underwent arthroscopic rotator cuff repair in the lateral decubitus position with upper extremity traction and postoperative intravenous patient-controlled analgesia. Both were considered low risk by conventional evaluation, with Wells pulmonary embolism scores of zero and routine preoperative investigations performed about four weeks before surgery showing no abnormalities. Despite the absence of typical respiratory symptoms, mild oxygen desaturation prompted D-dimer testing, which was markedly elevated in both cases. Computed tomography angiography confirmed asymptomatic pulmonary embolism in the early postoperative period, accompanied by deep vein thrombosis in the lower extremities. Both patients were successfully treated with direct oral anticoagulant therapy, leading to favorable outcomes without recurrence. These cases demonstrate that asymptomatic pulmonary embolism can occur early after arthroscopic rotator cuff repair, even in patients assessed as low risk. D-dimer testing in response to subtle oxygen desaturation may facilitate timely diagnosis, and vigilant monitoring with comprehensive risk assessment is essential to improve patient safety.

## Introduction

Arthroscopic rotator cuff repair (ARCR) has become widely established as a minimally invasive and commonly used surgical technique in the orthopaedic field. Postoperative venous thromboembolism (VTE) has a lower incidence compared to major lower extremity surgeries such as hip and knee joint procedures, with deep vein thrombosis (DVT) reported in less than 1% of cases and pulmonary embolism (PE) reported in 0.01 to 0.53% of cases [1-4]. Therefore, routine pharmacological prophylaxis is not recommended except for high-risk patients [1,5].

However, prospective cohort studies have reported a relatively high incidence of 5.7% when asymptomatic cases are included, indicating that the true incidence may vary depending on study design and patient population [5]. Furthermore, PE following arthroscopic shoulder surgery typically presents with symptoms such as dyspnea, chest pain, and palpitations before diagnosis, with symptom onset mainly occurring one to six weeks postoperatively [6,7].

Early-onset PE within one to three days postoperatively is uncommon, and there are virtually no reports of early-onset asymptomatic PE [7]. Such cases may be missed by conventional risk assessment and symptom-based diagnostic approaches, necessitating new strategies for early detection.

We report two cases of asymptomatic PE that developed early after ARCR, with a review of the literature.

## Case presentation

Case one

A 72-year-old female with a medical history of hypertension, hyperlipidemia, and osteoporosis presented with right shoulder pain following a fall. Physical examination revealed a body mass index of 26 and right shoulder pain. Preoperative assessment showed a Wells PE score of 0. Routine investigations including chest X-ray, electrocardiogram, and blood tests were performed 29 days before surgery and revealed no abnormalities.

ARCR was performed six months after injury. The patient was positioned in the lateral decubitus position with four kilograms of traction on the affected upper extremity. General anesthesia combined with interscalene block was administered, with intravenous patient-controlled analgesia (IV-PCA) used for postoperative pain management. Surgical procedures included joint capsule release, long head of biceps tenotomy, single-row repair of the subscapularis tendon, and suture bridge repair of the supraspinatus and infraspinatus tendons. Operative time was 91 minutes. No thromboprophylaxis measures were implemented.

**Figure 1:**
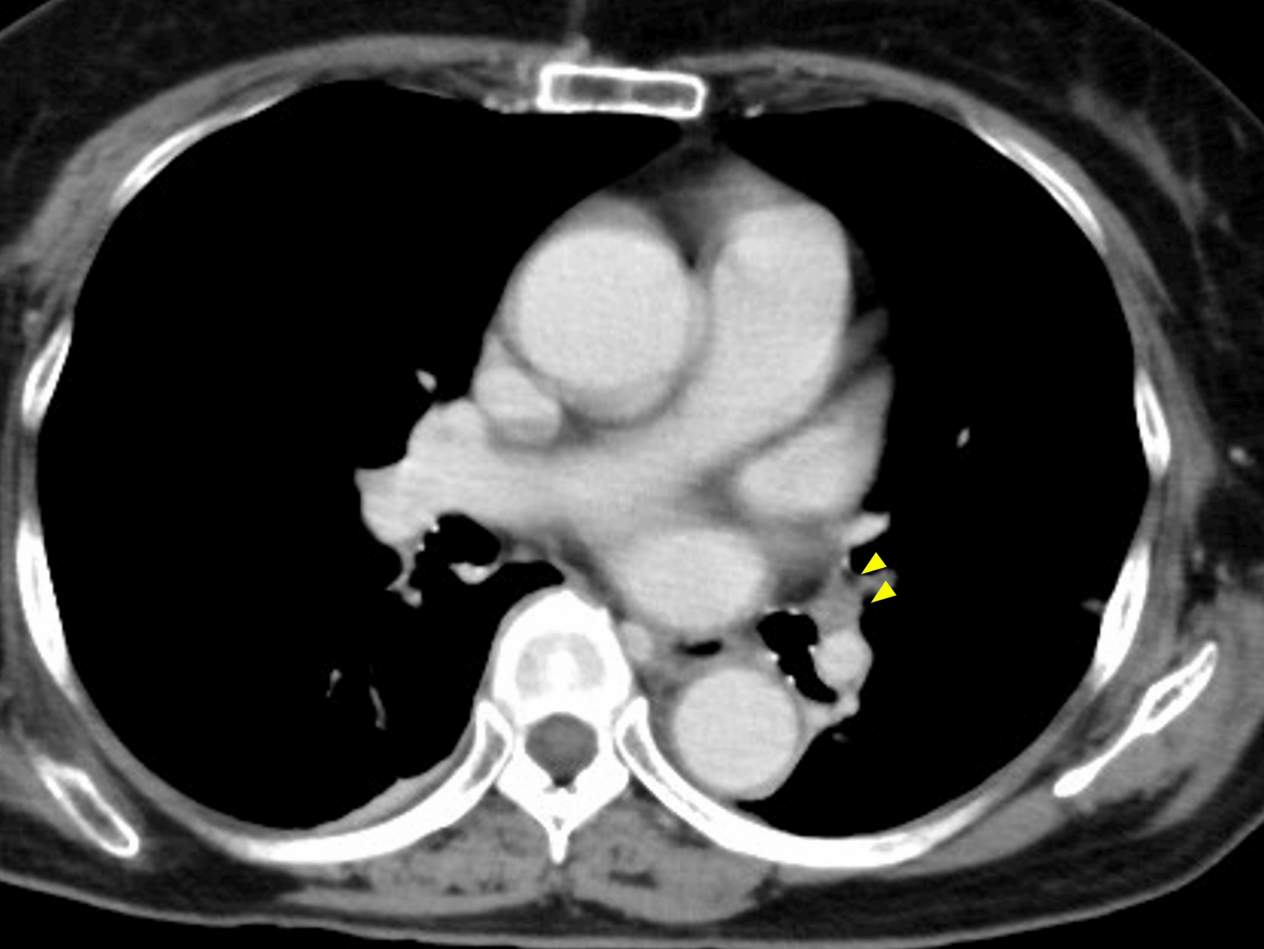
Computed tomography angiography findings in Case 1 Chest computed tomography angiography revealed blood flow interruption in the left pulmonary artery due to embolization (arrowhead).

**Figure 2:**
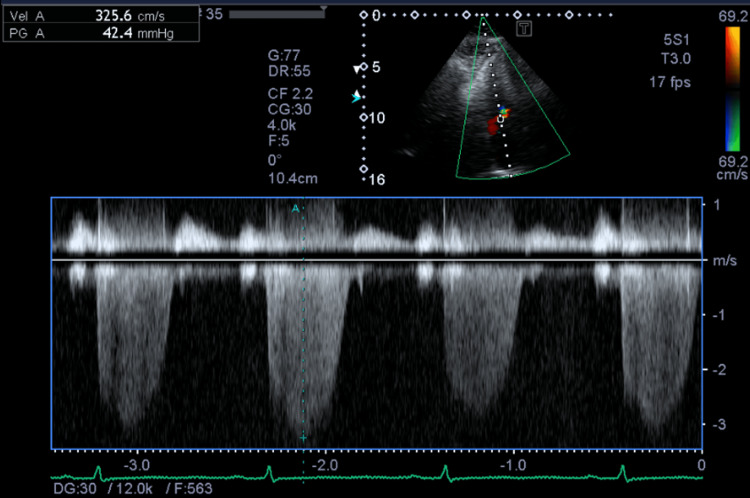
Echocardiographic Assessment of Pulmonary Hypertension The spectral Doppler trace demonstrates a peak velocity of 3.26 m/s, corresponding to a tricuspid regurgitation pressure gradient (TRPG) of 42.4 mmHg, indicating mild pulmonary hypertension.

Postoperatively, oxygen administration was discontinued three hours after surgery, and ambulation began four hours postoperatively. Oxygen saturation decreased from 96% to 92% at 12 hours postoperatively and further to the 70s at 18 hours, without dyspnea or respiratory distress. D-dimer was significantly elevated at 10.3 µg/mL (normal less than 1.0, Table 1), prompting computed tomography (CT) angiography, which diagnosed PE (Figure 1). Echocardiography revealed mild pulmonary hypertension with a tricuspid regurgitation pressure gradient (TRPG) of 42.4 mmHg (Figure 2). Lower extremity venous ultrasound confirmed acute bilateral soleus vein DVT, with right soleus vein thrombosis with 40 mm occlusion and left soleus vein thrombosis with 60 mm occlusion.

**Table 1:** Perioperative changes in D-dimer levels

Post-operation	Case 1 (µg/mL)	Case 2 (µg/mL)	Reference range
Day 1	10.3	42.8	<1.0
Week 1	-	9.3	<1.0
Week 2	3.8	1.7	<1.0
Month 1	0.5	0.5	<1.0
Month 3	0.1	0.9	<1.0

Direct oral anticoagulant (DOAC) therapy was immediately initiated, with rehabilitation starting after one week of bed rest. The patient was discharged four weeks postoperatively. Pulmonary hypertension findings resolved after three years of medication. At six months postoperatively, the Japanese Orthopaedic Association score improved from 74.5 to 94.5, and the Constant score improved from 46 to 78, demonstrating good functional improvement [8,9].

Case two

A 73-year-old female with a medical history of appendectomy, thoracic and lumbar vertebral fractures, and ankle osteoarthritis presented with right shoulder pain after injury. Physical examination revealed a body mass index of 24.4 and right shoulder pain. Preoperative assessment showed a Wells PE score of 0. Routine investigations including chest X-ray, electrocardiogram, and blood tests were performed 33 days before surgery and revealed no abnormalities.

ARCR was performed 17 months after injury. Positioning, anesthesia, and pain management were identical to case one. Surgical procedures included single-row repair of the subscapularis, supraspinatus, and infraspinatus tendons. Operative time was 77 minutes, with no thromboprophylaxis administered.

**Figure 3:**
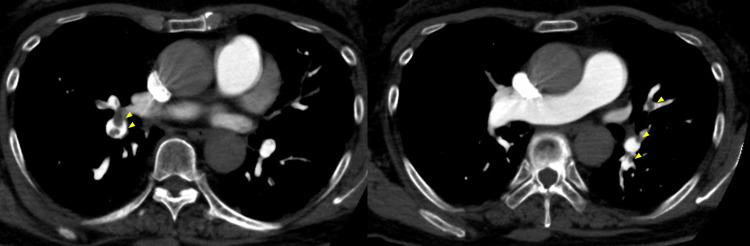
Computed tomography angiography findings in Case 2 Chest computed tomography angiography demonstrated multiple sites of blood flow interruption in bilateral pulmonary arteries (arrowheads).

Postoperatively, oxygen administration was discontinued three hours after surgery, but saturation dropped to the 80s without dyspnea, requiring continued oxygen. At 20 hours, oxygen saturation again remained in the 80s after discontinuation, still without dyspnea. Despite the absence of dyspnea, oxygen saturation remained in the 80s when oxygen was discontinued at 20 hours postoperatively. Although no obvious symptoms were present, D-dimer was elevated at 42.8 µg/mL (Table 1), prompting CT angiography which diagnosed PE (Figure 3). Lower extremity venous ultrasound revealed right soleus vein DVT with 35 mm occlusion.

Following heparin induction therapy, the patient was transitioned to DOAC therapy. She was discharged four weeks postoperatively, with medication discontinued at three months. At six months postoperatively, the Japanese Orthopaedic Association score improved from 68 to 81, and the Constant score improved from 40 to 64, showing functional improvement [8,9].

## Discussion

In both cases, PE developed within 24 hours postoperatively and was asymptomatic without clear respiratory symptoms. Because most reported PE cases following shoulder arthroscopy occurred later than three days after surgery, we regarded events within two days postoperatively as “early-onset” PE [7]. Common factors in both cases included lateral decubitus position surgery with upper extremity traction, IV-PCA for pain management, and elderly female patients. Early diagnosis was possible through D-dimer measurement triggered by mild oxygen saturation decline, and both cases showed favorable outcomes with DOAC therapy.

Risk factors for VTE after ARCR reported in the literature include obesity with body mass index greater than or equal to 30 kilograms per square meter [10,11,3], hypertension [10], male gender [11], operative time greater than or equal to 80 minutes [11,3], American Society of Anesthesiologists score of three or higher [11,3], diabetes [10,3], history of thrombotic disease [11,3], and intraoperative positioning including lateral decubitus or traction [12-14].

**Figure 4:**
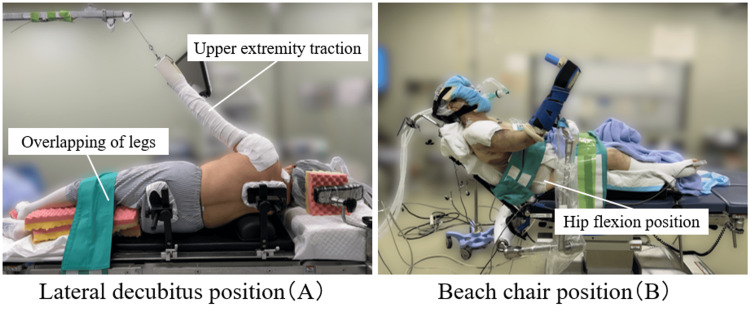
Surgical positioning for arthroscopic rotator cuff repair and associated thromboembolism risks (A) Lateral decubitus position demonstrating upper extremity traction with patient positioning that may compromise lower extremity venous return due to leg overlapping and positioning and potential compression. This position has been associated with increased risk of venous thromboembolism (VTE). (B) Beach chair position, which is considered relatively low risk for VTE compared to lateral decubitus positioning; however, excessive hip flexion in this position may potentially increase the risk of lower extremity deep vein thrombosis (DVT).

Regarding surgical position, venous thromboembolism events have been reported in association with lateral decubitus positioning and/or traction (Figure 4A) [12-14]. By contrast, a large series (15,033 cases) performed in the beach-chair position did not identify statistically significant patient- or surgery-related risk factors; all procedures were conducted in the beach-chair position [14]. However, caution is warranted because marked hip flexion may increase the risk of lower-extremity thrombosis (Figure 4B). In our case, acute thrombosis developed in the soleus vein of the contralateral limb (right leg in left lateral decubitus position). Although venous stasis in the dependent limb is a well-recognized risk, thrombosis can also occur in the soleus vein due to its anatomical propensity for blood stasis, and the laterality in our patient may have been incidental rather than position-related.

As demonstrated in our cases, even patients assessed as low risk by conventional risk evaluation may have increased VTE risk when lateral decubitus surgery is added, suggesting the need for more careful postoperative management when this position is selected.

As a diagnostic strategy, D-dimer elevation functions as a warning sign for PE and serves as a useful indicator for implementing CT pulmonary angiography [5]. Although postoperative D-dimer levels can increase due to non-thrombotic causes, the marked elevations observed in our cases greatly exceeded the typical range of postoperative variability, supporting their clinical significance. Our cases were in the low-risk group with a Wells PE score of 0 and did not meet conventional VTE high-risk criteria. However, the combination of factors such as lateral decubitus surgery and IV-PCA use demonstrated that patients who appear low risk may still have a risk of PE development. After ARCR, PE symptoms may be masked by respiratory depression from IV-PCA and patient tolerance to hypoxemia [5]. Therefore, routine D-dimer measurement for early postoperative oxygen saturation decline, regardless of symptoms, may enable early detection of asymptomatic PE.

For prevention, mechanical prophylaxis, including intermittent pneumatic compression devices and elastic stockings, is recommended as a reasonable, safe, and cost-effective option [5]. Improved pain management is also an important preventive measure. Recently, continuous brachial plexus block for postoperative analgesia has gained attention and shows promise as an effective alternative to general anesthesia plus IV-PCA [15,16]. Using local or regional anesthesia may avoid respiratory depression and potentially reduce VTE risk [15,16].

Regarding treatment selection, upper extremity DVT should be treated with the same anticoagulation regimen as lower extremity DVT [5]. In our cases, rivaroxaban was used in case one and edoxaban in case two, both showing favorable outcomes. DOACs have better medication compliance and lower bleeding risk compared to conventional heparin-warfarin therapy, making them suitable for outpatient treatment [5]. Treatment duration should continue for at least three months, and if unprovoked or permanent risk factors exist, should be extended beyond three months unless bleeding risk is high [5]. In our cases, treatment for three years in case one and three months in case two achieved pulmonary hypertension improvement and recurrence prevention [5], reflecting individualized clinical judgment.

## Conclusions

Although based on only two cases, we experienced two cases of asymptomatic PE developing early after ARCR. Both cases involved lateral decubitus surgery with IV-PCA use, enabling early diagnosis and treatment through D-dimer elevation. This report provides several important insights for clinical practice.

Even patients assessed as low risk by conventional risk evaluation may have asymptomatic PE development risk when lateral decubitus surgery and IV-PCA are used. D-dimer measurement is useful for diagnosis during early postoperative oxygen saturation decline, regardless of symptoms. Active investigation should be performed when D-dimer is elevated, even in patients assessed as low risk by the Wells PE score. A comprehensive approach including mechanical prophylaxis, improved pain management, and postoperative monitoring is important for optimal patient care.

Future research should establish comprehensive risk assessment including surgical position and appropriate postoperative monitoring systems for apparently low-risk cases. Early detection of asymptomatic PE is expected to improve safety after ARCR.
